# Chronic Patients' Activation and Its Association with Stress, Anxiety, Depression, and Quality of Life: A Survey in Southeast Iran

**DOI:** 10.1155/2021/6614566

**Published:** 2021-03-25

**Authors:** Mohammad Ali Zakeri, Mahlagha Dehghan, Fatemeh Ghaedi-Heidari, Maryam Zakeri, Gholamreza Bazmandegan

**Affiliations:** ^1^Non-Communicable Diseases Research Center, Rafsanjan University of Medical Sciences, Rafsanjan, Iran; ^2^Social Determinants of Health Research Center, Rafsanjan University of Medical Sciences, Rafsanjan, Iran; ^3^Nursing Research Center, Kerman University of Medical Sciences, Kerman, Iran; ^4^Faculty of Nursing and Midwifery, Isfahan University of Medical Sciences, Isfahan, Iran; ^5^Physiology-Pharmacology Research Center, Research Institute of Basic Medical Sciences, Rafsanjan University of Medical Sciences, Rafsanjan, Iran; ^6^Clinical Research Development Unit, Ali-Ibn Abi-Talib Hospital, Rafsanjan University of Medical Sciences, Rafsanjan, Iran; ^7^Department of Family Medicine, Ali-Ibn Abi-Talib Hospital, School of Medicine, Rafsanjan University of Medical Sciences, Rafsanjan, Iran

## Abstract

A better perception of the factors associated with patient activation, as a way to improve self-management, is the most important step in planning patient-centered education for chronic disease management. Therefore, the present study is aimed at investigating the relationship between activation, stress, anxiety, depression, and quality of life (QOL) in patients with chronic diseases. This correlational study was performed on 293 chronic patients admitted to coronary care units (CCUs) in one of the hospitals in Rafsanjan. The Patient Activation Measure (PAM), Quality of Life-BREF (WHOQOL-BREF), and Depression, Anxiety, and Stress Scale-21 Items (DASS-21) were used to collect data. The data were then analyzed using SPSS 22. A significant positive correlation was observed between general QOL and PAM (*P* < 0.001). In addition, a significant negative correlation was found between PAM, stress (*P* = 0.032), and depression (*P* = 0.025). The results of multivariate linear regression indicated that only physical and psychological subscales of QOL significantly predicted PAM (*B* = 0.24; 95% confidence interval; *P* value < 0.05). Owing to the fact that some subscales of QOL have a determinant role in the PAM of chronic patients, healthcare providers are recommended to plan and implement the necessary interventions to improve the QOL and the health outcomes of chronic patients.

## 1. Introduction

The silent pandemic of chronic diseases, one of the biggest public health challenges worldwide, is gradually spreading to all countries [1]. Chronic diseases have a high mortality rate and impose a heavy burden on healthcare systems [2]. According to the Institute of Health Metrics and Evaluation (IHME), chronic diseases accounted for 72% of the causes of death in 2016 [1]. According to the World Health Organization (WHO), chronic diseases caused 73 percent of deaths and 60% of the global burden of disease in 2020. In addition, 79% of these deaths will occur in developing countries [3]. The most common chronic diseases, including cardiovascular disease, cancer, chronic obstructive pulmonary disease (COPD), and type 2 diabetes have common and preventable risk factors such as hypertension, weight gain, and high-risk behaviors such as poor nutrition, sedentary lifestyle, and smoking [4]. According to the IHME report, ischemic heart disease was the leading cause of all deaths in the world and Iran in 2017 [5].

Self-management is one way to reduce the burden of disease on both the patient and the healthcare system and to reduce mortality of patients with chronic disease, which is one of the important factors involved [2]. Patient activation is one of the methods used to improve self-management. Patient activation refers to the knowledge, skills, and confidence in controlling one's health [6], which reflects the patient's perception of his/her role in the healthcare and self-management program [7]. The concept of patient activation has been demonstrated to correlate with improved clinical outcomes, increased preventative care, and decreased healthcare-related cost. This concept refers to the actions and activities performed by the patient to improve his/her illness. Patients being activated to manage their conditions had a positive effect on self-management and improved health outcomes [8, 9]. Assessing patient activation after a life-threatening illness provides opportunities for healthcare providers to develop care plans tailored to the patients' needs before their discharges [10].

In the last two decades, various studies have examined different populations by using the patient activation method. These studies show that patient activation is one of the effective factors in improving health-related behaviors and outcomes in patients with chronic diseases [11–13]. Greene and Hibbard showed that activation of patients with chronic diseases is associated with reduced admission to the emergency department, obesity, and smoking. In addition, patient activation minimizes the chance of breast cancer diagnosis in screening tests and abnormal changes in blood tests such as cholesterol, triglyceride, and glycosylated hemoglobin A_1_C [11]. One study found that chronically ill patients with lower levels of activation imposed higher costs on healthcare systems [12]. Furthermore, the study performed on patients with COPD showed a significant negative correlation between patients' activation, current smoking, hospital anxiety and depression scores, and respiratory symptoms [13]. Another study on patients with multiple sclerosis showed that patient activation is positively correlated with self-efficacy and academic achievement, but it had no significant relationship with QOL [14]. One study showed that patient activation increased satisfaction with postoperative outcomes in patients with lumbar and cervical spine diseases [15].

These studies have used a variety of methodologies and have mainly focused on parameters related to specific diseases. The present study assumed that many patients suffered from more than one chronic disease, and it did not emphasize a specific chronic disease. In addition, a better perception of some important variables (such as mental status and QOL) associated with patient activation is the most important step in planning patient-centered education for chronic disease management. Therefore, the present study is aimed at investigating the relationship between patient activation, stress, anxiety, depression, and QOL in patients with chronic diseases.

## 2. Methods

### 2.1. Study Design and Participants

This cross-sectional correlational study was conducted on 293 chronic patients admitted to the Cardiac Care Unit (CCU) and medical wards in Ali Ibn Abitaleb Hospital of Rafsanjan. Data were collected from January to April 2019. The inclusion criteria were chronically ill patients aged more than 18 years old, without known psychological problems (depression, bipolar disorder) and acute cognitive disorders. Patients with visual and auditory processing disorders were excluded from the study.

### 2.2. Study Setting

Iran is a collection of people with diverse languages and cultures. In general, the growth of urbanization in Iran is increasing, and the population in all cities has not grown evenly, but mainly large cities have grown faster than small cities. Being in the vicinity of the desert, southern and southeastern cities of Iran are less developed and economically grown and have brought about more problems and challenges for patients. For this reason, the QOL of people living in southeastern cities of Iran is different from those living in other places [16].

### 2.3. Sample Size and Sampling

Based on studies by Green et al. [17] to determine the relationship between activation, anxiety, stress, depression, and QOL (*r* = 0.21) with 99% confidence and 90% test power, the sample size was considered 240 people according to the following formula:

(1) $$\begin{array}{c} \omega =\frac{1}{2}\text{Ln}\frac{1+r}{1-r},\\ n=\frac{{\left(Z_{1-\alpha /2}+Z_{1-\beta }\right)}^{2}}{{\left(\omega \right)}^{2}}+3. \end{array}$$

**Figure 1:**
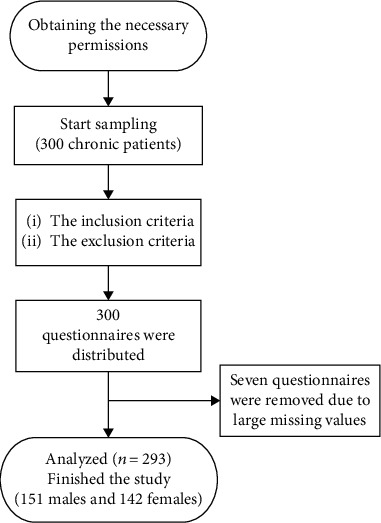
The study flowchart.

Concerning the conditions of chronic patients and the possibility of dropout, we examined 300 chronic patients according to inclusion criteria, and all of them wanted to participate in the study, so 300 questionnaires were collected. Out of these 300 questionnaires, seven questionnaires were excluded from the study due to deficiencies in completion, and finally, 293 were included in the analysis (Figure 1). The response rate was 97.66%.

### 2.4. Measurement

#### 2.4.1. Demographic Information

Demographic information of the participants included age, sex, body mass index (BMI), marital status, occupation, education level, income, number of hospital stays, presence of other diseases, drug use, and type of chronic disease.

#### 2.4.2. Patient Activation Measure-13

The American short form of Patient Activation Measure-13 (PAM-13) was developed by Hibbard et al. to examine self-management [6]. Activation therapy assesses the patient's knowledge, skills, beliefs, and confidence in health management and healthcare. This measure consists of 13 items on the Likert scale ranging from one (strongly disagree) to four (strongly agree). Answers are calculated based on the standard metric system converted from zero to 100 (zero = the lowest activation level, 100 = the highest level), and the score of the questionnaire varies from 0 to 100, with higher scores reflecting higher levels of patient activation. The validity of this questionnaire was obtained by using face and content validities. We used internal consistency and test-retest for the PAM questionnaire to assess reliability. The internal consistency was good (*α* = 0.88), and the intraclass correlation coefficient (ICC) was 0.96.

#### 2.4.3. WHOQOL-BREF

This questionnaire has been used by the WHO to measure the QOL of individuals in the last two weeks. This self-report questionnaire with 4 domains and 26 items examines the health status and QOL. The physical health domain, psychological domain, social relationship domain, and environmental domain are assessed in this questionnaire. It has also two questions for assessment of overall QOL and general health. The score of each item is on the scale value of 1 (never) to 5 (very high). Items 3, 4, and 25 are scored reversely. For this scale to be interpreted, the short version must be converted into a long version, and then, the QOL in each domain must be interpreted from zero to 100. It is noteworthy that the WHOQOL-BREF questionnaire does not allow a single QOL score. The higher scores indicate a better QOL. Nejat et al. confirmed the validity and reliability of the questionnaire in Iran in 2006. Cronbach's alpha coefficient of 0.78 was obtained for the questionnaire [18].

#### 2.4.4. Depression, Anxiety, and Stress Scale-21 Items (DASS-21)

The Depression, Anxiety, and Stress Scale-21 Items (DASS-21) was developed by Lovibond and Lovibond in 1995 to assess the psychological constructs of depression, anxiety, and stress [19]. The scale consists of 21 items with three subscales of depression, anxiety, and stress (each subscale includes seven items) on a four-point Likert scale (never/low/medium/high). The lowest score is zero, and the highest score is three. The final score of each is obtained through the sum of the scores of the related items. The final score of the subscales should be doubled. Samani and Joukar examined the validity and reliability of this scale in Iran and reported the retest validity to be 0.80, 0.76, and 0.77 for depression, anxiety, and stress, respectively. Cronbach's alpha coefficient was reported to be 0.81, 0.74, and 0.78, for depression, anxiety, and stress, respectively [20].

### 2.5. Data Collection and Analysis

After obtaining the necessary permissions, the researcher referred to the research settings and started sampling. Thus, the demographic information questionnaire, Patient Activation Measure-13, DASS, and WHOQOL-BREF were distributed among the eligible samples, who answered the questionnaires in the presence of the researcher. One researcher explained to the patients how to complete the questionnaires. According to the instructions for completing the questionnaire, patients were asked to complete the questions according to the last two weeks.

The data were analyzed by SPSS 22. Descriptive statistics (frequency, percentage, mean, and standard deviation) were used to describe the participants' characteristics. The Kolmogorov-Smirnov test was done to check the normal distribution of the quantitative data. Spearman and Pearson correlation coefficients were used to determine the correlation between the study quantitative variables. The independent *t*-test, Mann-Whitney *U*, analysis of variance, and Kruskal-Wallis tests were used to determine PAM-13 scores according to the qualitative variables. Multivariate linear regression with the enter method was used to identify the PAM-13 determinants. A significance level of 0.05 was considered.

### 2.6. Ethical Considerations

This research has a code of ethics No. IR.RUMS.REC.1397.109 from the University of Medical Sciences. Before sampling, informed written consent was taken from chronic patients, who were explained about the objectives of the study, confidentiality and anonymity of the information, and the voluntary participation in the study and voluntary withdrawal at any time. Participants with major anxiety, stress, and depression referred to a hospital psychiatrist for further evaluation. They also explained the consequences and problems of increasing anxiety, stress, and depression.

## 3. Results

The mean age of participants was 63.18 ± 13.44 years. The majority of the participants were male (51.5%), married (86.0%), and illiterate (50.2%) and had no history of hospital stay (37.2%) (Table 1).

The mean score of PAM-13 was 56.99 ± 15.32, which was greater than the midpoint of the questionnaire (score = 50). The mean scores of anxiety, stress, and depression were 23.79 ± 9.56, 25.35 ± 10.23, and 21.88 ± 10.27, respectively. The results showed that 20.9% (*n* = 61) and 62.0% (*n* = 181) of the participants had severe and extremely severe anxiety, respectively. 28.3% (*n* = 83), 23.5% (*n* = 69), and 21.8% (*n* = 64) of the participants had moderate, severe, and extremely severe stress, respectively. In addition, 21.2% (*n* = 62), 27.3% (*n* = 80), and 25.6% (*n* = 75) of the participants had moderate, severe, and extremely severe depression, respectively. No significant correlation was observed between anxiety, stress, and depression levels and PAM-13 score (*P* > 0.05) (Table 2).

The mean scores of physical, psychological, and social relationship and environmental subscales of QOL were 44.37 ± 17.50, 48.75 ± 13.33, 46.10 ± 20.17, and 48.96 ± 12.65, respectively. The mean score of general QOL was 47.05 ± 13.11.

The bivariate analysis showed that the mean score of PAM-13 was not significantly different according to the demographic and clinical characteristics of the participants (Table 1). A significant positive correlation was observed between all subscales of QOL except the social relationship subscale and PAM-13 (*P* < 0.05). In addition, a significant negative correlation was found between PAM-13, stress, and depression (*P* > 0.05) (Table 3). For further analysis, all variables with *P* value of <0.05 were included in the multiple linear regression analysis. The results of multivariate linear regression with the enter method indicated that only physical and psychological subscales of QOL predicted PAM-13 significantly (Table 4).

## 4. Discussion

The present study investigated the relationship between patient activation, stress, anxiety, depression, and QOL in chronically ill patients admitted to hospital. Based on the results of the study, the PAM score of patients was higher than average, which is not consistent with the results of inpatients and outpatients with chronic diseases in several studies. These studies showed that on average, 15% of the patients with chronic diseases had the lowest level of activation [21–24]. Methodological differences have played a role in this inconsistency. In this study, patients with chronic diseases were studied by a correlational method, while in other studies, for example, patients with certain types of chronic diseases such as acute coronary syndrome [21] or heart failure [24] were studied longitudinally. In addition, the postdischarge duration can play a role in patient activation. Some studies show that admitted patients or those discharged for less than a month have a higher level of activation [21, 25]. Owing to the fact that admitted patients have been studied in this study, a high activation score can be expected.

Many studies have shown that the QOL of chronic patients is negatively associated with depression and anxiety, which was also reflected in the present study, which showed a negative relationship between QOL, depression, and anxiety in cancer patients [26]. Stress also affected the QOL of cancer patients [27]. These results have also been seen in patients on hemodialysis [28] and with coronary heart disease [29]. Therefore, nurses and physicians, the main caregivers of patients, should pay attention to stress, anxiety, and depression in patients that can be effective in their QOL.

According to the results of the present study, a low PAM-13 score was associated with a high level of stress and depression; the association was statistically significant. In line with the current study, Magnezi et al., Blakemore et al., and Ahn et al. studied patients referred to primary care clinics [30], the older adults with chronic diseases [31], and patients with osteoarthritis [32], respectively, and found a negative correlation between depression and PAM.

In addition, the Pearson correlation test in the present study showed that a low PAM score was associated with a significant decrease in general QOL and its subscales except social relationship. However, only physical and psychological subscales of QOL significantly predicted the PAM-13. Thus, people better in some subscales of QOL may have higher levels of activation and, consequently, more capacity to participate in self-care behaviors [33, 34]. Erskine et al., who studied patients with acute coronary syndrome, obtained similar results [21]. Since the present study is cross-sectional, the causal relationship between the variables cannot be interpreted. However, depressed patients with lower QOL appear to have lower levels of activation and less involvement in self-management behaviors. Magnezi et al. showed that PAM scores correlated positively with a total Short Form-12 Health Survey (SF-12) score. Magnezi et al. pointed to a cycle in which depressed patients experienced a sense of helplessness and loss of QOL, which in turn is associated with less activation [32]. Therefore, healthcare providers, especially nurses, are expected to design and implement appropriate interventions, for example, psychological and pharmacological interventions [29] and patient support program [35] to reduce the symptoms of depression and improve the QOL in patients with chronic diseases, so they can play an effective role in increasing patient activation. These interventions ultimately reduce the admission rate of patients [36].

This study had several limitations: longitudinal studies are recommended to identify the causes of PAM change in patients with chronic diseases. Sampling was performed among admitted patients. Therefore, the generalization of results to outpatients should be done with caution. The self-report results of the patients may not always reflect a valid level of psychological impact, anxiety, stress, and depression of the chronic patients. Therefore, the social desirability bias may affect the results. The specific condition of chronic patients and the long course of treatment can affect the variables examined in these patients. In addition, a large number of questions can also affect patients' answers, which should be used with caution in interpreting the results.

## 5. Conclusion

According to the results of the present study, PAM is less associated with increased stress and depression and decreased some subscales of QOL in patients with chronic diseases. Only the physical and psychological subscales of QOL significantly predicted the level of PAM, so healthcare providers are recommended to plan and implement appropriate interventions to improve the QOL and the health-related outcomes.

## Figures and Tables

**Table 1:** Demographic and clinical characteristics of the participants (*N* = 293).

Variables	Mean (SD)	Patient Activation Measure-13	
		Spearman correlation coefficient	*P* value
Age (yr.)	63.18 (13.44)	-0.04	0.44
Body mass index	22.41 (3.28)	0.01	0.88
	*N* (%)	Statistical test	*P* value
Gender			
Male	151 (51.50)	*t* = −1.01	0.31
Female	142 (48.50)		
Marital status			
Married	252 (86.0)	*t* = −1.80	0.06
Unmarried+divorce	41 (14.0)		
Educational level			
Illiterate	147 (50.2)	*F* = 0.24	0.87
<diploma	83 (28.3)		
Diploma	43 (14.7)		
Academic	20 (6.8)		
Employment status			
Employed	133 (45.4)	*F* = 0.02	0.98
Unemployed	106 (36.2)		
Retired	54 (18.4)		
Income (million riyal)			
<0.5	113 (38.6)	*H* = 2.35	0.50
0.5–1	70 (23.9)		
1–2	79 (27.0)		
>2	31 (10.6)		
History of hospital stay (no.)			
0	109 (37.2)	*F* = 1.01	0.40
1	40 (13.7)		
2	42 (14.3)		
3	36 (12.3)		
>4	66 (22.5)		
Other illnesses			
Yes	186 (63.5)	*t* = −0.30	0.76
No	107 (36.5)		
Drug use			
Yes	98 (33.4)	*Z* = −1.58	0.11
No	195 (66.6)		
Diagnosis			
IHD	62 (21.2)	*F* = 1.55	0.18
Diabetes	55 (18.8)		
Hypertension	54 (18.4)		
CHF	45 (15.4)		
COPD	41 (14.0)		
Other^†^	33 (11.3)		

Data were presented numerically (%). *t* = independent *t*-test; *Z* = Mann-Whitney *U* test; *H* = Kruskal Wallis test; *F* = analysis of variance test; IHD = ischemic heart disease; CHF = congestive heart failure; COPD = chronic obstructive pulmonary disease. ^†^Chronic kidney disease, multiple sclerosis, rheumatoid arthritis, and cancer.

**Table 2:** The patient activation measure scores among chronic patients with different levels of anxiety, stress, and depression (*n* = 293).

Variables	Level	*N* (%)	Patient Activation Measure-13	
			Statistical test	*P* value
Anxiety	Normal	6 (2.1)	*F* = 0.83	0.73
	Mild	13 (4.5)		
	Moderate	31 (10.6)		
	Severe	61 (20.9)		
	Extremely severe	181 (62.0)		

Stress	Normal	44 (15.0)	*F* = 1.10	0.33
	Mild	33 (11.3)		
	Moderate	83 (28.3)		
	Severe	69 (23.5)		
	Extremely severe	64 (21.8)		

Depression	Normal	41 (14.0)	*F* = 1.08	0.35
	Mild	35 (11.9)		
	Moderate	62 (21.2)		
	Severe	80 (27.3)		
	Extremely severe	75 (25.6)		

Data were presented numerically (%).

**Table 3:** Correlation among the anxiety, stress, depression, quality of life, and patient activation measure in chronic patients (*n* = 293).

Variable	1	2	3	4	5	6	7	8
(1) Anxiety	1							
(2) Stress	0.72^∗∗^	1						
(3) Depression	0.67^∗∗^	0.64^∗∗^	1					
(4) Physical health subscale of QOL	-0.36^∗∗^	-0.32^∗∗^	-0.39^∗∗^	1				
(5) Psychological subscale of QOL	-0.16^∗∗^	-0.11	-0.22^∗∗^	0.53^∗∗^	1			
(6) Social relationship subscale of QOL	-0.26^∗∗^	-0.2^∗∗^	-0.32^∗∗^	0.54^∗∗^	0.58^∗∗^	1		
(7) Environmental subscale of QOL	-0.25^∗∗^	-0.18^∗∗^	-0.3^∗∗^	0.49^∗∗^	0.66^∗∗^	0.61^∗∗^	1	
(8) General QOL	-0.3^∗∗^	-0.26^∗∗^	-0.38^∗∗^	0.8^∗∗^	0.82^∗∗^	0.86^∗∗^	0.89^∗∗^	1
(9) PAM	-0.11	-0.13^∗^	-0.13^∗^	0.22^∗∗^	0.22^∗∗^	0.11	0.20^∗∗^	0.22^∗∗^

Data were presented as Pearson's correlation coefficient. ^∗^*P* < 0.05; ^∗∗^*P* < 0.01. QOL = quality of life.

**Table 4:** Predictors of patient activation measure scores by multiple linear regression analysis.

Predictors of Patient Activation Measure-13 scores	Unstandardized coefficients			Standardized coefficients	*t*	*P* value
	*B*	Std. error	95% CI for *B*	Beta		
Constant	44.92	5.11			8.78	<0.001
Stress	-0.09	0.11	-0.32–0.13	-0.06	-0.84	0.40
Depression	-0.03	0.12	-0.25–0.20	-0.02	-0.22	0.82
Physical health subscale of QOL	0.21	0.10	0.02–0.41	0.24	2.14	0.03
Psychological subscale of QOL	0.28	0.12	0.04–0.52	0.24	2.28	0.02
Environmental subscale of QOL	0.22	0.13	-0.04–0.48	0.18	1.65	0.10
General QOL	-0.39	0.24	-0.87–0.08	-0.34	-1.64	0.10

CI = confidence interval; QOL = quality of life.

## Data Availability

The data used to support the findings of this study are included within the article.
